# Isolated sternal fracture after low-energy trauma in a geriatric patient: a case report

**DOI:** 10.1186/s12245-022-00437-1

**Published:** 2022-07-29

**Authors:** Joni J. Sairanen, Otso Arponen

**Affiliations:** 1 Emergency Department, Tampere University Hospital, Tampere, Finland; 2 Department of Radiology, Tampere University Hospital, Tampere, Finland; 3 Faculty of Medicine and Health Technology, Tampere University, Tampere, Finland

**Keywords:** Sternal fracture, Trauma imaging, Falls, Osteoporosis, Case report

## Abstract

**Background:**

Falls are a common cause of emergency department (ED) visits for the older population. If osteoporosis is present, even falls from standing height can lead to unusual fractures normally associated with high-energy trauma. In this report, we analyze a rare case of an isolated sternal fracture with an unusual mechanism of injury. Our discussion aims to improve care for older adults with fall-related fractures.

**Case presentation:**

An 86-year-old female presented in the ED of our hospital with a complaint of chest pain. She recalled a fall at home the previous day and described how her fist was impacted between the floor and her chest. A physical examination revealed local tenderness in the mid-chest. A lateral chest x-ray indicated a sternal fracture, and a chest computed tomography scan ruled out concomitant injuries. There were no acute changes on her electrocardiogram (ECG). Conservative outpatient treatment was started, and referrals were made with a recommendation to initiate fall prevention measures and osteoporosis screening in primary health care.

**Conclusions:**

Geriatric patients can present in the ED with a rare sternal fracture even after only a minor chest trauma. Appropriate imaging and an ECG are warranted to exclude life-threatening additional injuries. An in-depth physical examination and an understanding of the exact mechanism of injury are important to avoid missing fractures in unexpected locations. Modern ED physicians could have an important role in the secondary prevention of fall-related fractures for geriatric patients.

## Background

Of all trauma admissions in the emergency department (ED), only 0.33–2.1% involve a sternal fracture (SF) due to blunt chest trauma [1, 2]. Motor vehicle accidents account for 83–84% of all SFs, while only 10–13% of SFs are due to falls, usually from height [3, 4]. Direct chest trauma is not the only possible mechanism of injury (MOI); a fall on the back could cause an SF due to flexion-compression [5]. Additional skeletal or visceral injuries are present in approximately 80% of patients with an SF due to blunt chest trauma [1, 2]. An isolated SF is diagnosed in the absence of concomitant injuries, and it can be described as a relatively mild injury [3, 6].

Eighty-seven percent of all fractures in persons older than 65 years are caused by falls [7]. The risk of fractures is markedly increased in patients with osteoporosis, which is both underdiagnosed and undertreated at the population level [8]. Potential risk factors for future falls and fractures should be identified and managed appropriately [7]. Even when a patient is discharged from the ED to go home, ED physicians could initiate secondary prevention by pinpointing the most obvious problems (e.g., inappropriate medication, poor vision or balance, orthostatic hypotension, frailty) and referring the patient to primary health care and osteoporosis screening.

## Case presentation

An 86-year-old female who lived alone at home arrived in the ED of our hospital and complained of chest pain that worsened with inspiration. She stated that the pain had started after a fall at home. Her medical records revealed a history of mild Alzheimer’s disease and hypertension. A photocopy of a recent ambulance report was found, according to which paramedics had responded to a chest pain call at her apartment the previous day. A rib fracture had been suspected and the patient was advised to seek medical attention in primary health care, but she arrived in the hospital ED instead.

The patient was in no apparent pain or distress. She was able to walk unassisted, although the overall impression suggested frailty. Respiration rate was 17/min, oxygen saturation 97% on room air, pulse 58/min, and blood pressure 156/72 mmHg. Troponin T concentration was within normal limits, and hemoglobin level (13.7 g/dL) was normal. An electrocardiogram (ECG) showed sinus rhythm with a prolonged PR interval (.218 s) and a bifascicular block, which were also present on her previous ECGs and appeared unchanged.

A physical examination revealed local pain in the sternum, but there were no external signs of trauma to the patient’s head, neck, or upper body. The rib cage was stable and nontender. Lung sounds were normal on auscultation. A systolic grade 2 murmur was noted. The cervical, thoracic, and lumbar spine were stable and nontender. No focal neurological deficits were noted.

**Fig. 1:**
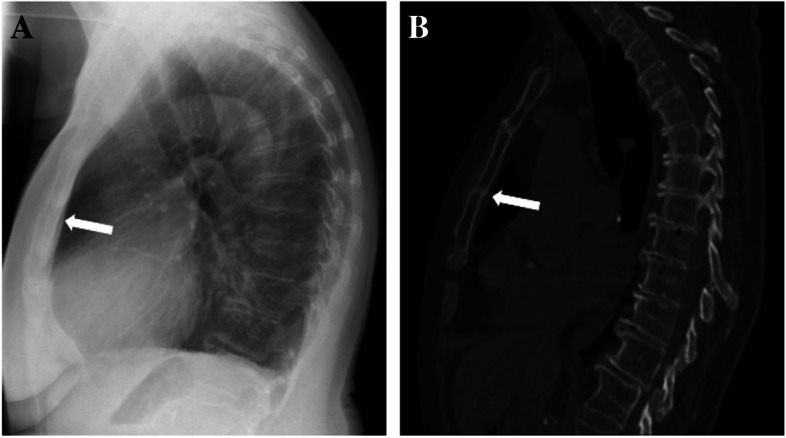
Isolated sternal fracture in a lateral chest x-ray (**A**) and a sagittal chest computed tomography view (**B**)

The patient underwent imaging (Fig. 1), and the initial radiology report of the chest x-ray (CXR) came back as normal. The CXR was reviewed in the ED. On the lateral view (A), a cortical breach in the body of the sternum was noted. There were no signs of newly displaced rib fractures, pneumothorax, or hemothorax. A computed tomography scan of the chest (B) confirmed the diagnosis of an isolated SF. No traumatic damage to major vessels or the heart could be discerned, but the ascending aorta was dilated (45 mm). Cortical thinning and loss of bony trabeculae were present, suggestive of osteopenia.

Upon further questioning, the patient gave a more precise description of the fall. She recalled how she fell forward, trapping her right fist between the floor and her chest. We believe this was an accurate account of the incident, despite the patient’s problems with short-term memory. The MOI is unusual but plausible. The patient denied having had episodes of syncope or transient loss of consciousness, and she did not use alcohol or any drugs that could have contributed to the fall.

Conservative treatment was started for isolated SF. The exact cause of the fall remained obscure, but frailty was interpreted as a risk factor for recurrent falls and fractures in the future. The patient’s relatives were informed, and the patient was discharged with prescriptions for acetaminophen and a vitamin D and calcium supplement. Written instructions were given to book a follow-up appointment with a primary care physician who could perform an osteoporosis workup and a comprehensive geriatric evaluation with the aim of preventing future falls. A cardiology referral was made due to the conduction abnormalities on the ECG, the systolic murmur, and the dilated ascending aorta.

## Discussion and conclusions

The thoracic cavity houses many of our vital organs and vessels. Significant chest trauma indicates imaging to diagnose or rule out not only skeletal fractures but also severe concomitant injuries (e.g., pulmonary contusion, pneumothorax, and mediastinal hematoma) [1, 6, 9]. The possibility of cardiac contusion must be kept in mind: patients with new arrhythmias, hypotension, or ST-segment abnormalities should be monitored in the hospital [10]. Patients with isolated SF very rarely require surgical treatment, and their prognosis is generally favorable [11]. Based on level II evidence, they can be safely discharged home if there are no other indications for hospitalization (e.g., comorbidities, need for parenteral pain medication, or lack of social support) [4]. Conservative treatment of SF involves rest and analgesia [11]. Passive reduction and corset fixation could be needed in patients with displaced SF [11].

The SF of our patient was noticed only upon review of the CXR in the ED – possibly because the radiologist who compiled the initial radiology report did not have the complete clinical picture (the precise MOI was unknown at the time of the imaging request). It has been estimated that missed fractures account for up to 79.7% of all diagnostic errors in the ED [12]. Detailed history and all relevant clinical findings should be disclosed in imaging requests because they help in choosing the most appropriate imaging modality [13]. Better imaging requests could also reduce the possibility of radiograph misinterpretation [13]; this is particularly the case in blunt chest trauma, where the CXR has a poor screening performance with only 45% sensitivity and 78% negative predictive value for injury [14].

The role of ED in the treatment of fractures need not be limited to acute care. In a Danish study by Jantzen et al. [15], all ED patients older than 65 years with a low-energy Colles’ fracture were systematically referred for osteoporosis screening. Sixty-five percent of the examined patients were diagnosed with osteoporosis, and almost all of them eventually received treatment with anti-osteoporosis drugs. Similarly, an ED physician could refer selected geriatric patients with any fall-related fractures to primary health care for comprehensive geriatric assessment and osteoporosis screening.

Fall-related fractures in older people are frequently encountered in the ED. Osteoporosis, a common and undertreated disease, predisposes patients to all types of fracture, including the rare SF. Radiologists’ work could be made easier by disclosing detailed history and all relevant clinical findings in imaging requests. The modern ED physician could have an important role in the secondary prevention of fall-related fractures.

## Data Availability

All data generated or analyzed during this study are included in this published article.

## Abbreviations

ED: Emergency department
SF: Sternal fracture
MOI: Mechanism of injury
ECG: Electrocardiogram
CXR: Chest x-ray
